# The genome sequence of the Miller, *Acronicta leporina* (Linnaeus, 1758)

**DOI:** 10.12688/wellcomeopenres.18889.1

**Published:** 2023-02-02

**Authors:** Douglas Boyes, University of Oxford and Wytham Woods Genome Acquisition Lab, Darwin Tree of Life Barcoding collective, Wellcome Sanger Institute Tree of Life programme, Wellcome Sanger Institute Scientific Operations: DNA Pipelines collective, Tree of Life Core Informatics collective, Peter W.H. Holland, Darwin Tree of Life Consortium

**Affiliations:** 1 UK Centre for Ecology and Hydrology, Wallingford, Oxfordshire, UK; 2 Department of Biology, University of Oxford, Oxford, Oxfordshire, UK

**Keywords:** Acronicta leporina, the Miller, genome sequence, chromosomal, Lepidoptera

## Abstract

We present a genome assembly from an individual female *Acronicta leporina* (the Miller; Arthropoda; Insecta; Lepidoptera; Noctuidae). The genome sequence is 466 megabases in span. Most of the assembly is scaffolded into 32 chromosomal pseudomolecules, including the W and Z sex chromosomes. The mitochondrial genome has also been assembled and is 15.4 kilobases in length.

## Species taxonomy

Eukaryota; Metazoa; Ecdysozoa; Arthropoda; Hexapoda; Insecta; Pterygota; Neoptera; Endopterygota; Lepidoptera; Glossata; Ditrysia; Noctuoidea; Noctuidae; Acronictinae; *Acronicta*; *Acronicta leporina* (Linnaeus, 1758) (NCBI:txid987862).

## Background

*Acronicta leporina*, described by Linnaeus in 1758, is a medium-sized noctuid moth (wingspan 35–45 mm) found across Europe. In the UK, the moth is known by the common name ‘the Miller’, although this name can be confusing as it is also used for different species of moth in other countries, including the army cutworm *Euxoa auxiliaris* in the USA.

Records of *A. leporina* range from north of the Arctic Circle in Scandinavia to the Mediterranean coast, with some putative records from East Asia ( GBIF Secretariat, 2023). In the UK, the moth is commonest in southern and central England, particularly in woodland and parkland habitats ( Randle *et al.*, 2019). Although many species of moths have been declining in abundance in the UK, *A. leporina* has shown a marked increase since 1970 ( Randle *et al*., 2019). Changes in abundance probably also occurred before recording was standardized, since in the 19th century it was considered a rarity ( Newman, 1869).

The wing pattern of the adult moth is distinctive, with a delicate pattern of black crescent marks on a pale background; the ground colour varies from white to grey and shows regional differences. Melanic and very dark grey forms have been recorded, with speculation that they have been subject to natural selection for camouflage in regions suffering from industrial pollution ( West, 1999). In the UK, the adult moth has a single generation per year with adults on the wing in June and July. The larvae feed on leaves of birch, alder or willow through late summer and autumn, before tunnelling into soft wood and pupating; the moth overwinters as a pupa, sometimes remaining in this life cycle stage for two winters ( Skinner & Wilson, 2009). The larva is easily recognisable due to a profusion of extraordinarily long lime green or yellowish hairs swirling away from its body; it has been described as “looking for all the world like a green Persian cat” ( Allan, 1948). A particularly unusual feature is the asymmetrical pattern of hairs: those on one side of the body generally curl forward, while those on the opposite side curl backwards ( South, 1961).

A genome sequence of *A. leporina* will facilitate studies of adaptation to specific food plants, formation of larval hairs, and convergent evolution of melanism in Lepidoptera.

## Genome sequence report

**Figure 1.**
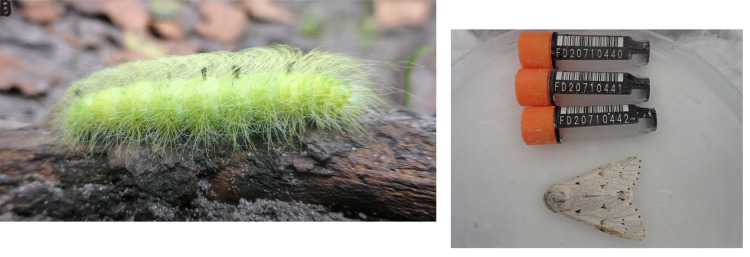
**a**) The larva of *Acronicta leporina.* Photograph by Amada44. **b**) Photograph of the *Acronicta leporina* (ilAcrLepo1) specimen used for genome sequencing.

The genome was sequenced from one female *Acronicta leporina* ( Figure 1b) collected from Wytham Woods (latitude 51.77, longitude –1.34). A total of 45-fold coverage in Pacific Biosciences single-molecule HiFi long reads was generated. Primary assembly contigs were scaffolded with chromosome conformation Hi-C data. Manual assembly curation corrected five missing or mis-joins and removed two haplotypic duplications, reducing the scaffold number by 7.89%, and increasing the scaffold N50 by 4.15%.

**Figure 2.**
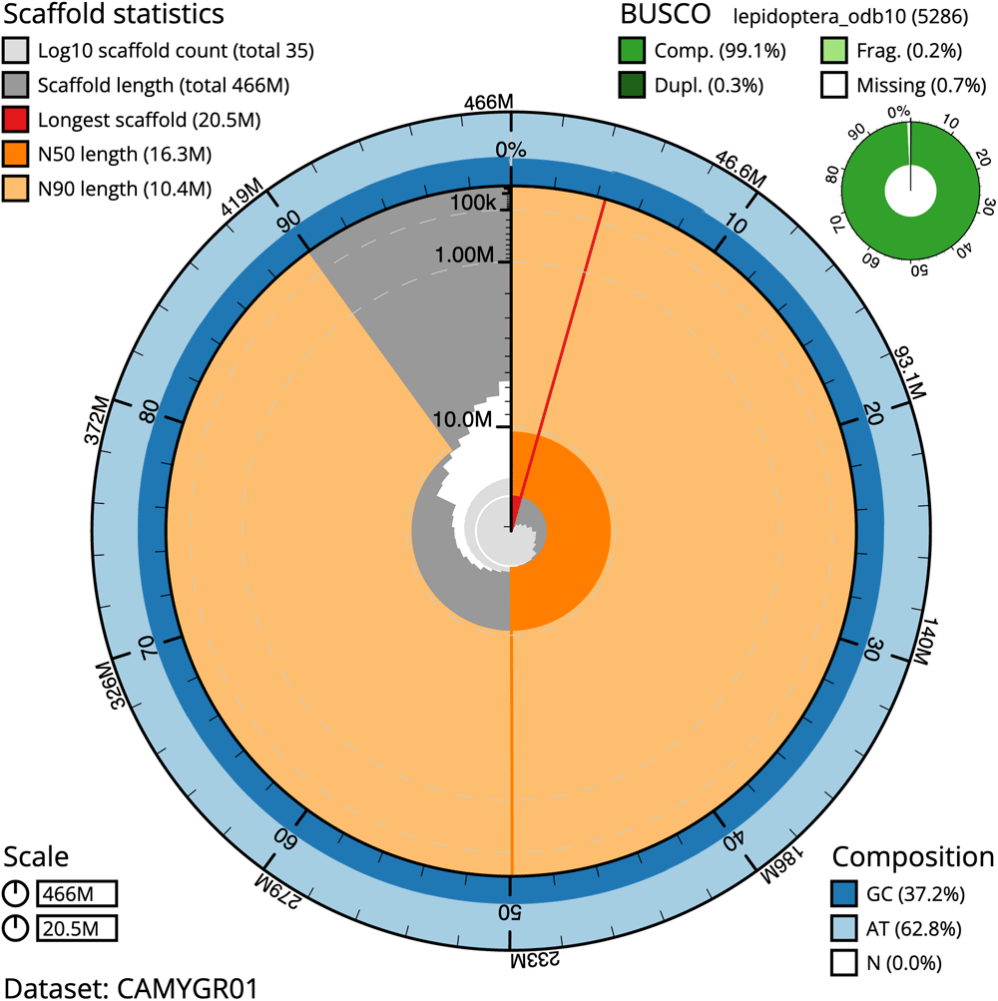
Genome assembly of *Acronicta leporina*, ilAcrLepo1.1: metrics. The BlobToolKit Snailplot shows N50 metrics and BUSCO gene completeness. The main plot is divided into 1,000 size-ordered bins around the circumference with each bin representing 0.1% of the 465,591,081 bp assembly. The distribution of scaffold lengths is shown in dark grey with the plot radius scaled to the longest scaffold present in the assembly (20,521,448 bp, shown in red). Orange and pale-orange arcs show the N50 and N90 scaffold lengths (16,347,223 and 10,385,229 bp), respectively. The pale grey spiral shows the cumulative scaffold count on a log scale with white scale lines showing successive orders of magnitude. The blue and pale-blue area around the outside of the plot shows the distribution of GC, AT and N percentages in the same bins as the inner plot. A summary of complete, fragmented, duplicated and missing BUSCO genes in the lepidoptera_odb10 set is shown in the top right. An interactive version of this figure is available at https://blobtoolkit.genomehubs.org/view/ilAcrLepo1.1/dataset/CAMYGR01/snail.

The final assembly has a total length of 465.6 Mb in 35 sequence scaffolds with a scaffold N50 of 16.3 Mb ( Table 1). Most (99.98%) of the assembly sequence was assigned to 32 chromosomal-level scaffolds, representing 30 autosomes and the W and Z sex chromosomes. Chromosome-scale scaffolds confirmed by the Hi-C data are named in order of size ( Figure 2– Figure 5; Table 2). The assembly has a BUSCO v5.3.2 ( Manni *et al.*, 2021) completeness of 99.1% using the lepidoptera_odb10 reference set. While not fully phased, the assembly deposited is of one haplotype. Contigs corresponding to the second haplotype have also been deposited.

**Table 1.** Genome data for *Acronicta leporina*, ilAcrLepo1.1.

Project accession data		
Assembly identifier	ilAcrLepo1.1	
Species	*Acronicta leporina*	
Specimen	ilAcrLepo1	
NCBI taxonomy ID	987862	
BioProject	PRJEB56406	
BioSample ID	SAMEA10979192	
Isolate information	female, thorax (PacBio sequencing), head (Hi-C sequencing)	
Assembly metrics *		*Benchmark*
Consensus quality (QV)	66.4	*≥ 50*
*k*-mer completeness	100%	*≥ 95%*
BUSCO **	C:99.1%[S:98.8%,D:0.3%], F:0.2%,M:0.7%,n:5286	*C ≥ 95%*
Percentage of assembly mapped to chromosomes	99.98%	*≥ 95%*
Sex chromosomes	ZW	*localised homologous pairs*
Organelles	Mitochondrial genome assembled	*complete single alleles*
Raw data accessions		
PacificBiosciences SEQUEL II	ERR10357391	
Hi-C Illumina	ERR10313053	
Genome assembly		
Assembly accession	GCA_947256265.1	
*Accession of alternate haplotype*	GCA_947256475.1	
Span (Mb)	465.6	
Number of contigs	73	
Contig N50 length (Mb)	9.3	
Number of scaffolds	35	
Scaffold N50 length (Mb)	16.3	
Longest scaffold (Mb)	20.5	

* Assembly metric benchmarks are adapted from column VGP-2020 of “Table 1: Proposed standards and metrics for defining genome assembly quality” from ( Rhie *et al.*, 2021). ** BUSCO scores based on the lepidoptera_odb10 BUSCO set using v5.3.2. C = complete [S = single copy, D = duplicated], F = fragmented, M = missing, n = number of orthologues in comparison. A full set of BUSCO scores is available at https://blobtoolkit.genomehubs.org/view/ilAcrLepo1.1/dataset/CAMYGR01/busco.

**Figure 3.**
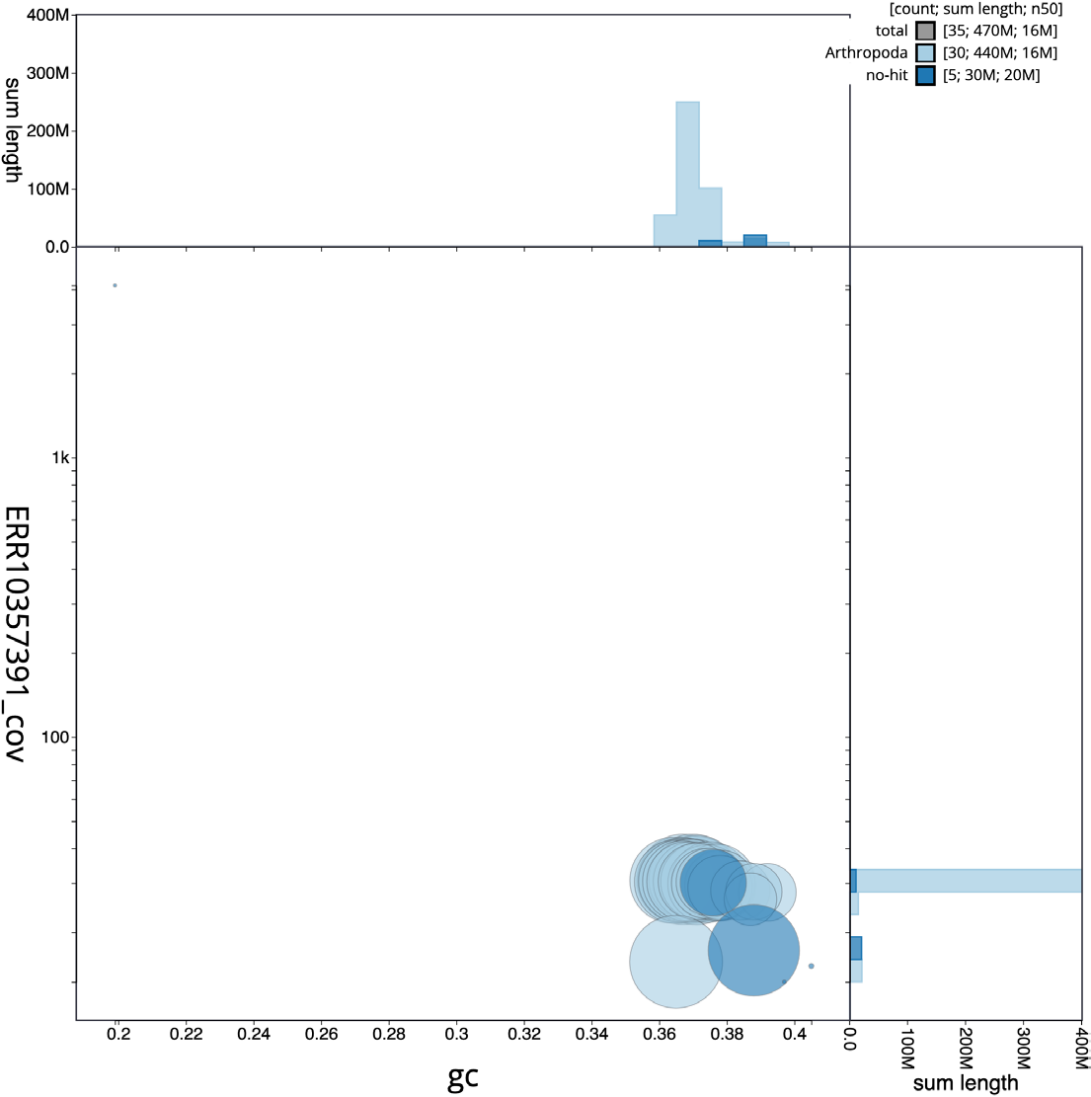
Genome assembly of *Acronicta leporina*, ilAcrLepo1.1: GC coverage. BlobToolKit GC-coverage plot. Scaffolds are coloured by phylum. Circles are sized in proportion to scaffold length. Histograms show the distribution of scaffold length sum along each axis. An interactive version of this figure is available at https://blobtoolkit.genomehubs.org/view/ilAcrLepo1.1/dataset/CAMYGR01/blob.

**Figure 4.**
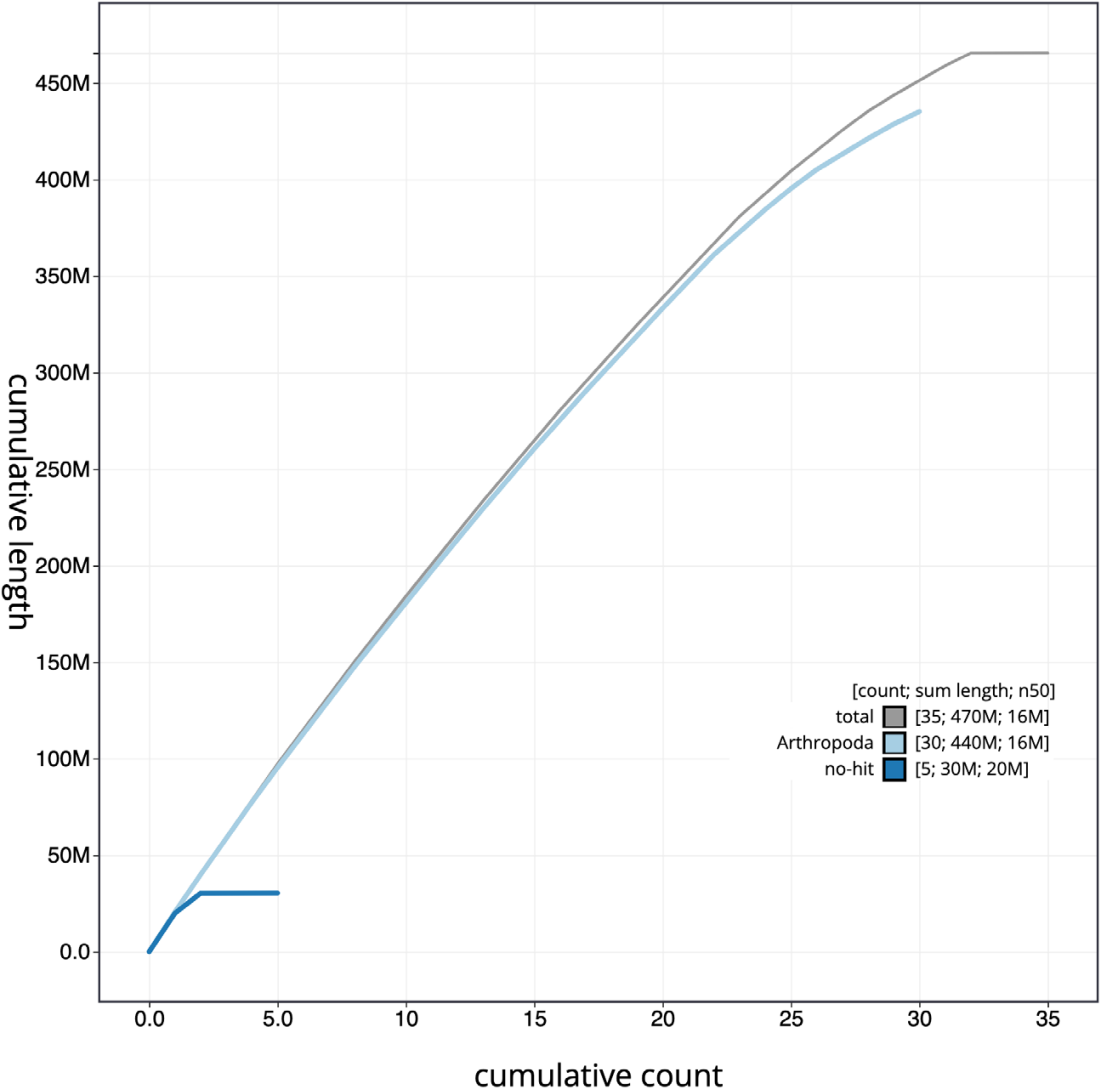
Genome assembly of *Acronicta leporina*, ilAcrLepo1.1: cumulative sequence. BlobToolKit cumulative sequence plot. The grey line shows cumulative length for all scaffolds. Coloured lines show cumulative lengths of scaffolds assigned to each phylum using the buscogenes taxrule. An interactive version of this figure is available at https://blobtoolkit.genomehubs.org/view/ilAcrLepo1.1/dataset/CAMYGR01/cumulative.

**Figure 5.**
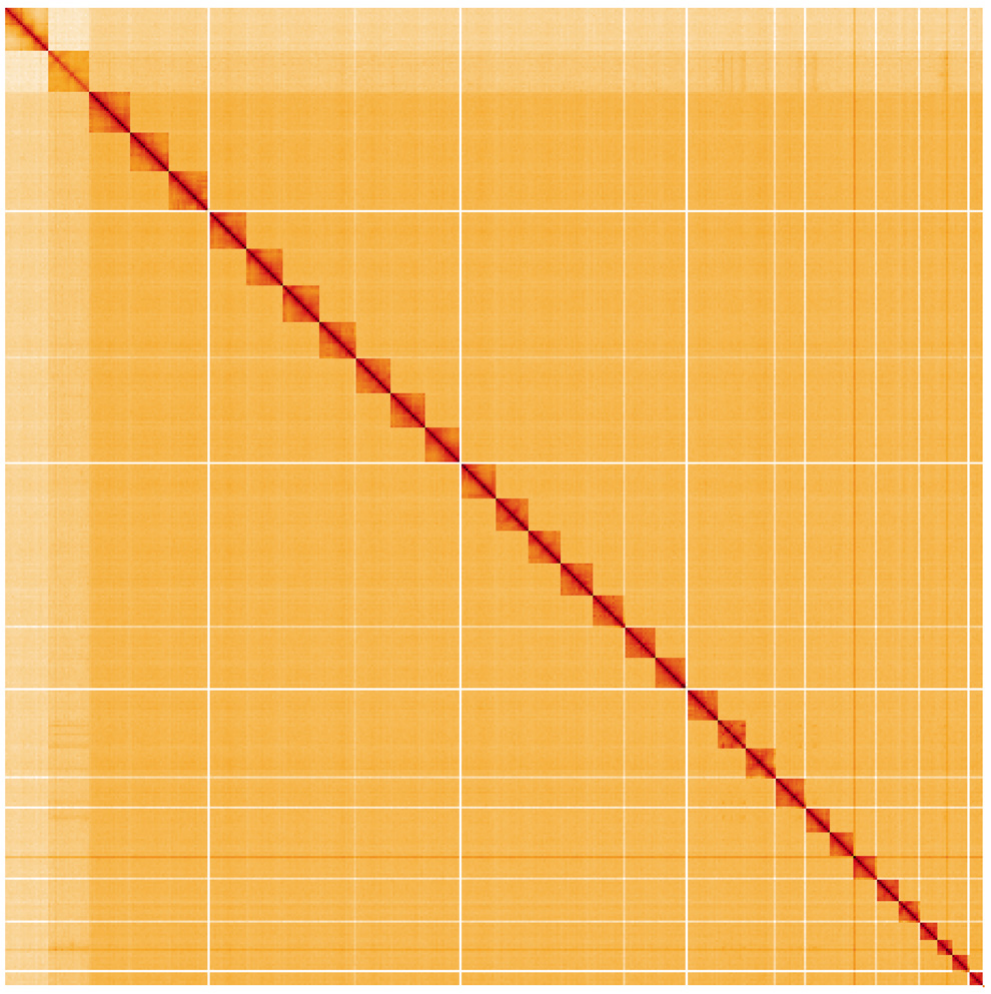
Genome assembly of *Acronicta leporina*, ilAcrLepo1.1: Hi-C contact map. Hi-C contact map of the ilAcrLepo1.1 assembly, visualised using HiGlass. Chromosomes are shown in order of size from left to right and top to bottom. An interactive version of this figure may be viewed at https://genome-note-higlass.tol.sanger.ac.uk/l/?d=AVoKtLp9R8C1Z4rRvP8ctQ.

**Table 2.** Chromosomal pseudomolecules in the genome assembly of *Acronicta leporina*, ilAcrLepo1.

INSDC accession	Chromosome	Size (Mb)	GC%
OX366512.1	1	19.38	36.7
OX366513.1	2	19	37
OX366514.1	3	18.54	37.1
OX366515.1	4	18	37.1
OX366516.1	5	17.55	36.6
OX366517.1	6	17.49	36.4
OX366518.1	7	17.22	36.7
OX366519.1	8	16.61	36.6
OX366520.1	9	16.5	36.5
OX366521.1	10	16.46	37
OX366522.1	11	16.35	36.9
OX366523.1	12	15.87	36.6
OX366524.1	13	15.7	36.9
OX366525.1	14	15.41	36.7
OX366526.1	15	14.98	37
OX366527.1	16	14.78	36.8
OX366528.1	17	14.47	37.2
OX366529.1	18	14.29	37.5
OX366530.1	19	14.23	37.4
OX366531.1	20	13.97	37.7
OX366532.1	21	13.81	37.1
OX366533.1	22	11.81	37.8
OX366534.1	23	11.62	37.4
OX366535.1	24	10.79	37.5
OX366536.1	25	10.39	37.6
OX366537.1	26	9.8	37.8
OX366538.1	27	8.29	38.4
OX366539.1	28	7.82	39.2
OX366540.1	29	7.48	38.8
OX366541.1	30	6.5	38.7
OX366511.1	W	19.87	38.8
OX366510.1	Z	20.52	36.5
OX366542.1	MT	0.02	20.2

## Methods

### Sample acquisition and nucleic acid extraction

A single *A. leporina* (ilAcrLepo1) was collected from Wytham Woods, Oxfordshire (biological vice-county: Berkshire), UK (latitude 51.77, longitude –1.34) on 16 June 2021 using a light trap. The specimen was collected and identified by Douglas Boyes (University of Oxford) and snap-frozen on dry ice. The sex of the individual was not determined morphologically at the time of collection, but was inferred retrospectively from the genome assembly.

DNA was extracted at the Tree of Life laboratory, Wellcome Sanger Institute (WSI). The ilAcrLepo1 sample was weighed and dissected on dry ice with head tissue set aside for Hi-C sequencing. Thorax tissue was cryogenically disrupted to a fine powder using a Covaris cryoPREP Automated Dry Pulveriser, receiving multiple impacts. High molecular weight (HMW) DNA was extracted using the Qiagen MagAttract HMW DNA extraction kit. HMW DNA was sheared into an average fragment size of 12–20 kb in a Megaruptor 3 system with speed setting 30. Sheared DNA was purified by solid-phase reversible immobilisation using AMPure PB beads with a 1.8X ratio of beads to sample to remove the shorter fragments and concentrate the DNA sample. The concentration of the sheared and purified DNA was assessed using a Nanodrop spectrophotometer and Qubit Fluorometer and Qubit dsDNA High Sensitivity Assay kit. Fragment size distribution was evaluated by running the sample on the FemtoPulse system.

### Sequencing

Pacific Biosciences HiFi circular consensus DNA sequencing libraries were constructed according to the manufacturers’ instructions. DNA sequencing was performed by the Scientific Operations core at the WSI on Pacific Biosciences SEQUEL II (HiFi) instrument. Hi-C data were also generated from head tissue of ilAcrLepo1 using the Arima v2 kit and sequenced on the Illumina NovaSeq 6000 instrument.

### Genome assembly

Assembly was carried out with Hifiasm ( Cheng *et al.*, 2021) and haplotypic duplication was identified and removed with purge_dups ( Guan *et al.*, 2020). The assembly was scaffolded with Hi-C data ( Rao *et al.*, 2014) using YaHS ( Zhou *et al.*, 2022). The assembly was checked for contamination as described previously ( Howe *et al.*, 2021). Manual curation was performed using HiGlass ( Kerpedjiev *et al.*, 2018) and Pretext ( Harry, 2022). The mitochondrial genome was assembled using MitoHiFi ( Uliano-Silva *et al.*, 2022), which performed annotation using MitoFinder ( Allio *et al.*, 2020). The genome was analysed and BUSCO scores were generated within the BlobToolKit environment ( Challis *et al.*, 2020). Table 3 contains a list of all software tool versions used, where appropriate.

**Table 3.** Software tools and versions used.

Software tool	Version	Source
BlobToolKit	3.5.0	Challis *et al.*, 2020
Hifiasm	0.16.1-r375	Cheng *et al.*, 2021
HiGlass	1.11.6	Kerpedjiev *et al.*, 2018
MitoHiFi	2	Uliano-Silva *et al.*, 2022
PretextView	0.2	Harry, 2022
purge_dups	1.2.3	Guan *et al.*, 2020
YaHS	yahs-1.1.91eebc2	Zhou *et al.*, 2022

### Ethics/compliance issues

The materials that have contributed to this genome note have been supplied by a Darwin Tree of Life Partner. The submission of materials by a Darwin Tree of Life Partner is subject to the Darwin Tree of Life Project Sampling Code of Practice. By agreeing with and signing up to the Sampling Code of Practice, the Darwin Tree of Life Partner agrees they will meet the legal and ethical requirements and standards set out within this document in respect of all samples acquired for, and supplied to, the Darwin Tree of Life Project. Each transfer of samples is further undertaken according to a Research Collaboration Agreement or Material Transfer Agreement entered into by the Darwin Tree of Life Partner, Genome Research Limited (operating as the Wellcome Sanger Institute), and in some circumstances other Darwin Tree of Life collaborators.

## Data Availability

European Nucleotide Archive: *Acronicta leporina* (the miller). Accession number PRJEB56406; https://identifiers.org/ena.embl/PRJEB56406. ( Wellcome Sanger Institute, 2022) The genome sequence is released openly for reuse. The *Acronicta leporina* genome sequencing initiative is part of the Darwin Tree of Life (DToL) project. All raw sequence data and the assembly have been deposited in INSDC databases. The genome will be annotated using available RNA-Seq data and presented through the Ensembl pipeline at the European Bioinformatics Institute. Raw data and assembly accession identifiers are reported in Table 1.

## Author information

Members of the University of Oxford and Wytham Woods Genome Acquisition Lab are listed here: https://doi.org/10.5281/zenodo.4789928.

Members of the Darwin Tree of Life Barcoding collective are listed here: https://doi.org/10.5281/zenodo.4893703.

Members of the Wellcome Sanger Institute Tree of Life programme are listed here: https://doi.org/10.5281/zenodo.4783585.

Members of Wellcome Sanger Institute Scientific Operations: DNA Pipelines collective are listed here: https://doi.org/10.5281/zenodo.4790455.

Members of the Tree of Life Core Informatics collective are listed here: https://doi.org/10.5281/zenodo.5013541.

Members of the Darwin Tree of Life Consortium are listed here: https://doi.org/10.5281/zenodo.4783558.
