# Supplementary material for: The genome sequence of the Miller, Acronicta leporina (Linnaeus, 1758)
Source: Wellcome Open Res. 2023 Feb 2;8:49. [Version 1] doi: 10.12688/wellcomeopenres.18889.1 (PMC10632593; doi:10.12688/wellcomeopenres.18889.1)
Supplement: Peer review history

# Reviewer report: Reviewer response for version 1

**DOI:** 10.21956/wellcomeopenres.20945.r68610

**Published:** 2023-11-08

**Authors:** Fan Song

**Affiliations:** 1 China Agricultural University, Beijing, China

This article presented a genome assembly of an individual female *Acronicta Leporina*, with a genome size of 466 Mb. Most of the assembly is scaffolded into 32 chromosomal pseudomolecules, including the W and Z sex chromosomes. Additionally, the mitochondrial genome has been assembled to a length of 15.4 kb. Here, I have some comments that may be useful in improving the quality of the paper.

1. The description of how to identify sex chromosomes is not present in the article. Please add more details about identify sex chromosomes methods.
2. It is suggested that the horizontal and vertical coordinates of Figure 5 could have a label showing the size of the genome and indicating which is the W and Z sex chromosome.
3. Please add information about the sequencing data coverage of Hi-C in the article.

Are sufficient details of methods and materials provided to allow replication by others?

Yes

Is the rationale for creating the dataset(s) clearly described?

Yes

Are the datasets clearly presented in a useable and accessible format?

Yes

Are the protocols appropriate and is the work technically sound?

Yes

Reviewer Expertise:

Insect genomics

I confirm that I have read this submission and believe that I have an appropriate level of expertise to confirm that it is of an acceptable scientific standard.

# Reviewer report: Reviewer response for version 1

**DOI:** 10.21956/wellcomeopenres.20945.r64491

**Published:** 2023-10-13

**Authors:** Fabrice Legeai

**Affiliations:** 1 IGEPP, INRAE, Rennes, France

This article is technically complete and provide a good and complete overview of the generation of the genome, achieved with standard and approved methods.

 My only concern is that the coverage of the  chromosomes which apparently points out the sex chromosomes Z and W is not reported in the  Table 2 (although attendant in the blobtoolkit interactive table).

Are sufficient details of methods and materials provided to allow replication by others?

Yes

Is the rationale for creating the dataset(s) clearly described?

Yes

Are the datasets clearly presented in a useable and accessible format?

Yes

Are the protocols appropriate and is the work technically sound?

Yes

Reviewer Expertise:

Bioinformatics, insect genomics

I confirm that I have read this submission and believe that I have an appropriate level of expertise to confirm that it is of an acceptable scientific standard.
